# New models of care in general practice for the youth mental health transition boundary

**DOI:** 10.3399/bjgpopen20X101133

**Published:** 2020-10-07

**Authors:** Rebecca Appleton, Faraz Mughal, Domenico Giacco, Helena Tuomainen, Catherine Winsper, Swaran P Singh

**Affiliations:** 1 Warwick Medical School, University of Warwick, Coventry, UK; 2 School of Primary, Community and Social Care, Keele University, Keele, UK; 3 Unit of Academic Primary Care, University of Warwick, Coventry, UK; 4 Warwick Medical School, University of Warwick, Coventry, UK; 5 R & I Department, Caludon Centre, Coventry and Warwickshire Partnership Trust, Coventry, UK; 6 Centre of Mental Health and Wellbeing, Warwick Medical School, University of Warwick, Warwick, Coventry, UK

**Keywords:** Mental health, Transition, Young people, General practitioners, Mental health services, General practice, Primary healthcare

## Setting the scene

Mental illness represents the highest proportion of disease burden for children and young people in the UK.^1^ However, despite this, young people can struggle to access timely and appropriate mental health care. One particular barrier to continuity of care occurs when young people reach the upper age limit (usually 18 years) of child and adolescent mental health services (CAMHS). If they require ongoing specialist support, their care should be transferred to an adult mental health service (AMHS), through a purposeful and planned transfer of care known as ‘transition’.

However, only around a quarter of young people transition to AMHS,^2^ and in the absence of specialist adult mental health care, GPs often become involved in the young person’s care ‘by default’.^3^ Although GPs become responsible for the young person’s care after they leave CAMHS, they may not have the necessary skills and resources to manage complex mental health difficulties in young people.

### The role of the GP in transition

The National Institute for Health and Care Excellence (NICE) transition guidance calls for a named GP to be part of the transition process, emphasising the role GPs can have in continuity of care.^4^ The guidance states that the person acting as the ‘named worker’ (who is responsible for coordinating transition for the young person) should proactively engage GPs in transition planning, allowing GPs to be involved in the young person’s mental health care at this crucial stage. At present, however, there is no known evidence to suggest that GPs are regularly involved in transition planning. This may be due to differing organisational cultures which prevent collaborative working; something which has been identified as a barrier to continuity of care between CAMHS and AMHS.^5^

Therefore, instead of a planned transfer of care, GPs become involved ‘by default’ and become responsible for both monitoring and prescribing medication to young people once they are discharged from CAMHS, in some cases without specialist support.^3^ This can result in young people being unable to continue their medication after leaving CAMHS, or feeling as though their need for continued medication had not been sufficiently reviewed.^6^

If the transition to AMHS were to be properly planned, GPs would be well placed to provide ongoing care for young people when they are discharged from specialist mental health services, as they may have longstanding relationships with young people and their families.^7^ This relational continuity has been shown to help engagement for patients discharged from an early intervention in psychosis service.^7^ Patients valued the ability to access local primary care services, especially when they had a trusting relationship with their GP.^7^

There is currently no evidence-based model regarding the involvement of general practice in transitional care. In this commentary, we outline three potential models and discuss the preliminary evidence for how these models can be beneficial in enhancing continuity of care for young people at the mental health transition boundary.

### 1) Improved training for GPs

GPs can find managing young people’s mental health problems challenging.^8^ For example, GPs have reported anxiety around managing young people in emotional distress, in part due to lack of medical education and clinical experience with young people who present with complex difficulties.^8^ Other training needs identified by GPs include prescribing psychotropic medication and communicating with young people.^9,10^ A recent trial in which GPs were trained in adolescent risk-taking behaviours, using a screening tool, and motivational interviewing improved the detection of health risk behaviours in young people.^11^ A similar approach aiming to improve GP knowledge about the specific challenges faced by young people at the CAMHS transition boundary may enhance GPs’ confidence in managing the care of these young people and improve mental health outcomes.

### 2) Collaborative care models

Despite a dearth of literature regarding shared care models in young people’s mental health in the UK, there is evidence to support their effectiveness in other vulnerable populations. A randomised controlled trial of collaborative care versus usual GP care found collaborative care resulted in a statistically significant improvement in older adults’ mental health.^12^ This model used a primary care mental health worker or an Improving Access to Psychological Therapies professional to provide the collaborative care, using a combination of active surveillance, telephone support, and symptom monitoring. Important information was shared with the patients’ GP and other mental health professionals to facilitate medication reviews or consultations regarding physical health needs.

A similar model could be implemented to support CAMHS leavers, which may ensure that young people are able to continue taking medication, and that they are able to receive regular medication reviews. Schraeder and Reid^13^ suggest a collaborative care model with a tiered approach, in which young people who have high symptom severity are transitioned to AMHS, and those with low symptom severity but a high risk of recurrence receive follow-up appointments to monitor their symptoms in primary care. This may be acceptable to young people, as both young people and their parents have reported feeling abandoned when CAMHS ends,^6^ and having follow-up appointments in primary care after discharge could alleviate anxiety around leaving CAMHS.

### 3) Mental health professionals in general practice

Guidance from NHS England released in 2018 encourages the placement of mental health professionals in primary care settings to facilitate access to care while reducing the impact of mental health consultations on GP workload.^14^ Patients have identified several benefits of visiting a mental health professional in a primary care setting including ease of access and improved communication between healthcare professionals.^15^

Several studies have evaluated the use of mental health nurses in primary care.^16,17^ Redeploying trained community psychiatry nurses into general practice across primary care networks has the potential to result in positive mental health patient outcomes,^16^ improved access to mental health care, shorter waiting times, and better integrated care.^17^ In addition, studies have reported reduced stigma for accessing mental health services within primary care.^17^

### Considerations for future research

Currently, there is a lack of research into interventions to improve continuity of care for young people in general practice after leaving CAMHS. There is an urgent need for future studies exploring potential models of care which could enhance mental health support for young people who do not meet eligibility criteria for AMHS. In particular, participatory research would focus on the needs of young people who do not transition to AMHS, and GPs‘ views on the types of interventions that would improve continuity of care. Little is also known about what type of support young people would like to receive from their general practice after leaving CAMHS. This should also be addressed in future studies to ensure interventions meet the needs of transition-aged youth.

## Conclusion

Only a quarter of young people transition to AMHS after leaving CAMHS, leaving the majority under the care of their GP after crossing the CAMHS–AMHS transition boundary. We know little about how GPs manage the care of these young people and with the rising numbers of young people needing CAMHS care, there is an urgent need to better understand how GPs can support young people with mental health problems once they reach the CAMHS boundary. Supporting GPs may involve establishing clear collaborative care arrangements with specialist services, incorporating mental health professionals into general practice, and providing appropriate training to support GPs to manage the needs of these young people. Future research should focus on the feasibility, acceptability, effectiveness, implementation, and sustainability of potential models within a primary care systems context, to inform guidelines for clinical service and pathway transformation.
